# Universal cervical cancer control through a right to health lens: refocusing national policy and programmes on underserved women

**DOI:** 10.1186/s12914-020-00237-9

**Published:** 2020-07-31

**Authors:** Katrina Perehudoff, Heleen Vermandere, Alex Williams, Sergio Bautista-Arredondo, Elien De Paepe, Sonia Dias, Ana Gama, Ines Keygnaert, Adhemar Longatto-Filho, Jose Ortiz, Elizaveta Padalko, Rui Manuel Reis, Nathalie Vanderheijden, Bernardo Vega, Bo Verberckmoes, Olivier Degomme

**Affiliations:** 1 International Centre for Reproductive Health, Department of Public Health & Primary Care, Ghent University, C. Heymanslaan 10 UZ/ICRH, 9000 Ghent, Belgium; 2 Dalla Lana School of Public Health, University of Toronto, Toronto, Canada; 3 Academic Network on Sexual and Reproductive Health & Rights Policy, Ghent, Belgium; 4 Instituto Nacional de Salud Pública, Cuernavaca, Mexico; 5 NOVA National School of Public Health, Public Health Research Centre, Universidade NOVA de Lisboa, Lisbon, Portugal; 6 Centre for Social Studies on Migration and Refugees, Ghent University, Ghent, Belgium; 7 Molecular Oncology Research Center, Barretos Cancer Hospital, Barretos, São Paulo, Brazil; 8 Medical Laboratory of Medical Investigation 14, Department of Pathology, Faculdade de Medicina, Universidade de São Paulo, São Paulo, Brazil; 9 Life and Health Sciences Research Institute, School of Medicine, University of Minho, Braga, Portugal; 10 ICVS/3B’s PT Government Associate Laboratory, Braga/Guimarães, Portugal; 11 Faculty of Medical Sciences of the University of Cuenca, Cuenca, Ecuador; 12 Department of Diagnostic Sciences, Ghent University, Ghent, Belgium; 13 Department Virology, Parasitology, Immunology, Ghent University, Ghent, Belgium

**Keywords:** Cervical cancer, Human papillomavirus, Sexual and reproductive health, Right to health, Human rights, Cancer screening, Cancer prevention, National cancer policy, HPV test

## Abstract

**Background:**

Cervical cancer claims 311,000 lives annually, and 90% of these deaths occur in low- and middle-income countries. Cervical cancer is a highly preventable and treatable disease, if detected through screening at an early stage. Governments have a responsibility to screen women for precancerous cervical lesions. Yet, national screening programmes overlook many poor women and those marginalised in society. Under-screened women (called hard-to-reach) experience a higher incidence of cervical cancer and elevated mortality rates compared to regularly-screened women. Such inequalities deprive hard-to-reach women of the full enjoyment of their right to sexual and reproductive health, as laid out in Article 12 of the International Covenant on Economic, Social and Cultural Rights and General Comment No. 22.

**Discussion:**

This article argues first for tailored and innovative national cervical cancer screening programmes (NCSP) grounded in human rights law, to close the disparity between women who are afforded screening and those who are not. Second, acknowledging socioeconomic disparities requires governments to adopt and refine universal cancer control through NCSPs aligned with human rights duties, including to reach all eligible women. Commonly reported- and chronically under-addressed- screening disparities relate to the *availability* of sufficient health facilities and human resources (example from Kenya), the *physical accessibility* of health services for rural and remote populations (example from Brazil), and the *accessibility of information* sensitive to cultural, ethnic, and linguistic barriers (example from Ecuador). Third, governments can adopt new technologies to overcome individual and structural barriers to cervical cancer screening. National cervical cancer screening programmes should tailor screening methods to under-screened women, bearing in mind that eliminating systemic discrimination may require committing greater resources to traditionally neglected groups.

**Conclusion:**

Governments have human rights obligations to refocus screening policies and programmes on women who are disproportionately affected by discrimination that impairs their full enjoyment of the right to sexual and reproductive health. National cervical cancer screening programmes that keep the right to health principles (above) central will be able to expand screening among low-income, isolated and other marginalised populations, but also women in general, who, for a variety of reasons, do not visit healthcare providers for regular screenings.

## Background

Cervical cancer is the fourth most common cancer in women, globally. Every two minutes a woman dies of cervical cancer around the world, resulting in 311,000 deaths annually [1]. Nine out of ten of these deaths occur in low- and middle-income countries (LMICs)- a fact that the World Health Organization (WHO) Director-General Dr. Tedros Adhanom Ghebreyesus labels ‘neither fair nor just’ [2]. *Why?* Cervical cancer is a highly preventable and treatable disease if detected at an early stage. Prevention and early detection is possible by checking for abnormalities in the cells of the cervix. Also the presence of the human papillomavirus (HPV) is an indication that one might be at risk for cervical cancer; especially the so called high-risk HPV types increase the risk of malignant lesions. Two of them, HPV 16 and 18, are found in over 70% of cervical cancer cases. These preventative measures have long hinged on a functioning health system, complete with trained gynaecologists, laboratory infrastructure, and vaccination and screening programs. All of these measures are necessary to work towards cervical cancer elimination, a global commitment in the 2013–2020 *Global Action Plan for the Prevention and Control of Noncommunicable Diseases*. The UN Population Fund has promised to support national health ministries in integrating this into existing reproductive health programmes [3].

Governments have a three-part responsibility in relation to cervical cancer over the course of a woman’s life: during her youth, to vaccinate against HPV; in midlife to screen for precancerous cervical lesions; and at all ages to treat cancer, if needed [4]. These times are crucial thresholds that can become a life saved or a life lost. Of these three moments, regular screening and follow-up of *all* at-risk women is the pinnacle of cervical cancer control. When under-screened, women experience a higher incidence of cervical cancer and elevated mortality rates compared to regularly-screened women [5]. Yet, crucial shortcomings in national cervical cancer screening programmes (NCSPs) mean they overlook many poor women and those marginalised in society by their age, ethnicity, disability, language, place of residence, and/or recent immigration status, among other factors [6]. Such inequalities harm women twice, first by making them more vulnerable to acquiring HPV infections, and again by depriving them of potentially lifesaving screening and early cancer detection. Ultimately, these women cannot enjoy their right to health, as laid out in Article 12 of the International Covenant on Economic, Social and Cultural Rights (ICESCR). The 2016 General Comment No. 22 is an authoritative explanation of governments’ obligations to realise the right to sexual and reproductive health (SRH) from Article 12 of the ICESCR.

At the core of this debate, is how NCSPs, especially those in LMICs, can effectively reach populations vulnerable to HPV infections through policy and practice. This article argues for tailored and innovative NCSPs grounded in human rights law to close the disparity between women who are afforded screening and those who are not. Human rights law has the potential to reorient social norms, political discourse, and government’s legal obligations towards the needs of under-served women and girls [7]. Some NCSPs may be framed around human rights law, such as the *Kenyan National Cancer Control Strategy 2017–2022* mentioned below. However, to the authors’ knowledge, no systematic study of NCSPs’ alignment with human rights law has been undertaken. Human rights-based screening programmes have the potential to make an important contribution to attaining, by 2030, a one-third reduction in premature mortality from non-communicable diseases (NCDs), such as cervical cancer (Sustainable Development Goal Target 3.4).

In the following sections of this paper, we first examine States’ human rights obligations towards cervical cancer control, including through NCSPs, as outlined in General Comment No. 22, and the need to address disparities. We then recommend that acknowledging socioeconomic disparities requires governments to adopt and refine universal cancer control through NCSPs aligned with human rights duties, including to reach all eligible women. Third, we advocate for governments in countries with disparities in cervical cancer incidence and mortality to adopt new technologies to overcome individual and structural barriers to cervical cancer screening.

## Discussion

### Cervical cancer control as part of the right to sexual and reproductive health

The right to the highest attainable standard of physical and mental health (‘right to health’) is embedded in numerous international treaties, including the most prominent, the ICESCR. In total, 169 national governments (or States) have ratified the ICESCR, and are therefore, legally obliged to realise the right to health. Due to ongoing and grave violations of people’s SRH, the UN Committee on Economic, Social and Cultural Rights (UN CESCR) drew attention to the ‘right to sexual and reproductive health’ (‘right to SRH’), a component of the right to health, in General Comment No. 22 (2016) [4]. This is a non-binding, yet highly authoritative, explanation of government obligations to realise the right to SRH.

Each State must use its own means and methods to realise the right to SRH with a maximum of its available resources, according to General Comment No. 22. This flexibility recognises that there is no ‘one-size-fits-all’ approach to SRH; instead, each government should use a tailored strategy to respond to local SRH needs and challenges within its own resources. Despite this flexibility, each State is under the immediate obligation “to eliminate discrimination against individuals and groups and to guarantee their equal right to SRH,” (4, para 34). In other words, States must immediately ensure that whatever their actions, they ensure equality and non-discrimination for all, and even “implement temporary special measures to overcome long-standing discrimination... and to eradicate conditions that perpetuate discrimination,” (4, para 36), where needed. Notably, the UN CESCR makes these legal obligations specific to reproductive cancers in the provision (emphasis added):

*States should aim to ensure universal access without discrimination for all individuals, including those from disadvantaged and marginalised groups, to a full range of quality SRH care, including... [the] prevention, diagnosis, and treatment of ...****reproductive cancers****... (4, para 45).*

States also have ‘core obligations’ under the right to SRH, which signify the basic minimum level that governments must achieve in order to give meaning to the right to SRH. Among these core obligations is the duty to “guarantee universal and equitable access to affordable, acceptable and quality SRH services, goods, and facilities, in particular for disadvantaged and marginalised groups” (4, para 49c), including “access to comprehensive education and information on SRH that are non-discriminatory, unbiased, evidence-based” and that are tailored to the capacities of children and adolescents (4, para 49f), and the provision of “medicines, equipment and technologies essential to SRH, including those based on the WHO Model List of Essential Medicines” (4, para 49 g). The HPV vaccine is one of these recommended medicines, based on the 2019 WHO Model List [8]. The WHO has also included an HPV DNA testing device on its list of essential in-vitro diagnostics for healthcare facilities with clinical laboratories [9]. When read together with States’ legal obligations (4, para 45), these core obligations can be understood to include non-discriminatory access to services, information, education, medicines, and technologies for the prevention and treatment of reproductive cancers.

Cervical cancer screening programmes should also be aligned with the AAAQ framework: Availability, Accessibility, Acceptability, and Quality of health goods and services necessary for the right to SRH (4, paras 11–21). Quality is of particular relevance to universal cervical cancer control. Ensuring quality services requires providing the HPV vaccine when it is most effective at preventing disease (i.e. before first sexual activity), screening for lesions as a scientific and medically appropriate measure for prevention and early detection, and providing assured quality cancer care in case of a cancer diagnosis.

### Screening that reaches every woman

In most high-income countries, screening is standard practice and guidelines target women most at risk of developing cervical cancer [10, 11]. By contrast, screening is much less common in LMICs due to its high cost and the limited health infrastructure [10, 11]. A study of six LMICs found those with absent or newly implemented screening guidelines had the lowest rates of crude and effective cervical cancer screening, with high cancer incidence and mortality, while countries with established guidelines had higher screening rates and lower disease burden [10]. Even NCSPs that are explicitly designed to reach all eligible women may still miss the most disadvantaged people. NCSPs should be designed with this challenge in mind. This requires prioritising the needs of the most hard-to-reach women if these programmes truly aspire to reduce health inequalities. Evidence shows that cervical cancer-related deaths drop to ≤2 women per 100,000 when screening (with a Pap test) is done every 3–5 years and reaches 70% of eligible women [5, 12]. Achieving- and exceeding- this goal requires a move towards population-based screening instead of opportunistic screening (where the latter reaches women already in contact with the healthcare system or women presenting symptoms of cervical abnormalities). Yet, many women around the world go unscreened despite the introduction of NCSPs [13].

Indeed, social inequalities are at the heart of many screening disparities. The examples presented below illustrate commonly reported- and chronically under-addressed- screening disparities relating to: the *availability* of sufficient health facilities and human resources (Kenya); the *physical accessibility* of health services for rural and remote populations (Brazil); and the *accessibility of information* sensitive to cultural, ethnic, and linguistic barriers (Ecuador). Still, there are a number of other, often overlapping, social disadvantages that impair women’s universal access to screening. Structural disadvantages that women face include difficulty registering with or navigating the health system, especially understanding one’s entitlement to care, and the cost and inconvenience of travelling to or the screening services themselves, among other issues [6, 14]. A woman can face a variety of complex personal and structural barriers that exacerbate her access to screening. Therefore, in the context of cervical cancer screening, hard-to-reach women are considered to be women aged 30 to 65, who are sexually active, and who, for various reasons, are not reached by screening services and consequently, are at higher risk for cervical cancer [6, 14].

The *availability* of physicians and gynaecologists, and an “adequate number of functioning health-care facilities... to provide the population with the fullest possible range of SRH care” (4, para 12), can be a root cause of inaccessible screening. For example, the *Kenyan National Cancer Control Strategy 2017–2022* foresees piloting a population-based screening program in “counties where comprehensive regional cancer centres are being planned” [15]. Indeed, the physician-to-population ratio- a proxy measure of the availability of trained healthcare providers- varies significantly between different Kenyan counties, from 1:143,000 in a hard-to-reach community to 1:21,000 in a community with three district hospitals [16]. Although this pilot screening program is an important (first) step, a rights-based approach will plan to take “deliberate, targeted, and concrete” measures to scale-up the number of health providers reaching women beyond the areas surrounding cancer care facilities (4, para 33). Ultimately, a more comprehensive approach will be needed in Kenya, where only 3.5% of eligible women report ever being screened [13]. Consequently, in Kenya- like much of sub-Saharan Africa- cervical cancer is the leading cause of cancer-related deaths among women [17].

The example of Brazil illustrates how crucial *physically accessible* screening services “within safe physical and geographic reach for all” (4, para 16) are so that women may receive timely care, reducing the incidence of cervical cancer. Brazil employs an opportunistic screening programme that has achieved disparate levels of coverage and cancer survival across the country. Fragmented screening means that women at risk or with early-stage cancer are missed and only present to health facilities in late stages when their chances of survival are lower [18]. In particular, ensuring women can reach health facilities with trained gynaecologists and quality-controlled laboratories has limited the reach of some screening programs. Disparate access in screening services has resulted in a decrease in cervical cancer-related mortality in the developed southern, southeastern, and midwestern regions of Brazil, while an increase in the less developed areas of the northern and northeastern regions is observed [19, 20]. Consequently, the screening benefits are not enjoyed equally by all women in Brazil. Aware of these disparities, some Brazilian cancer hospitals now use mobile units, complete with laboratories, to bring screening to women in remote locations. Between 2002 and 2012, these vehicles navigated difficult terrain without roads, animal herds, and water crossings via ferry to screen 174,605 women who were unlikely to have otherwise been tested for cervical cancer or its precursors [21].

Information and education about cervical cancer screening, diagnosis, and preventative treatment should be tailored and *accessible* to hard-to-reach women. These measures are consistent with the ‘core obligations’ of comprehensive education and information about how to prevent, diagnose and treat sexually-transmitted infections, such as HPV, and reproductive cancers (4, para 49f). For example, despite providing free-of-charge cervical cancer screening at health facilities, Ecuador has low national screening coverage (9–23% of eligible women) and a high incidence of cervical cancer (19 women per 100,000) [22, 23]. In order to improve this picture, attention should be paid to indigenous people’s needs as they constitute 1.1 million of the 16.4 million people in Ecuador, yet are often marginalised in health matters [24]. Indigenous women face multiple forms of discrimination when accessing screening provided in health centres: language is a barrier for non-Spanish speakers to access care and these women may experience judgement by healthcare providers [25, 26]. Individual barriers are also at play, such as gender norms, cultural customs, and a mistrust of Western medicine (that are possibly related to past mistreatment), which inhibit these women from undergoing a Pap test [26, 27]. Although these factors can have a chilling effect on women’s screening attendance, it is most telling that information promoting screening has failed to reach indigenous women. Some indigenous women report first learning about the Pap test when receiving primary care for their first pregnancy or after their first child [27]. A holistic public health and human rights approach requires that information about sexually-transmitted infections and reproductive cancer be *accessible* and “provided in a manner consistent with the needs of the individual and the community, taking into consideration, for example, age, gender, language ability, education level, disability, sexual orientation, gender identity, and intersex status” (4, para 18–19).

### Vaccination and screening

HPV vaccination is a promising strategy to prevent high-risk infections. The HPV vaccines are extremely effective at preventing infections by common high-risk HPV types [28, 29]. Yet HPV vaccination is most effective when given before women are exposed to the virus. This limitation means that women who are vaccinated before their first sexual activity will benefit most from HPV vaccination, not women who are already sexually active [5]. Eradicating HPV infections through vaccination is currently difficult because most immunisation programmes only target girls and women, while boys and men can also transmit the virus and develop HPV-related cancers [5]. Herd immunity will be more easily reached when all potential carriers are vaccinated. Based on the latest demography updates, HPV vaccination provides more health benefits and is more cost-effective than previously estimated [30].

The widescale implementation of the nonavalent vaccine – US Food and Drug Administration (FDA) approved in 2014 - will be another crucial step towards stopping HPV transmission. The vaccine offers protection to 9 genotypes, of which 7 oncogenic (versus 2 high-risk HPV types in the previously approved vaccines), and is clinically proven to prevent HPV-related diseases in both sexes [31].

However, HPV vaccines can only achieve these public health gains if they are available and affordable for women and health systems [4]. Many countries, some with a high burden of cervical cancer, experience significant lag time in implementing the HPV vaccine in national programmes despite its approval by the FDA over 10 years ago [32]. It is also important to remember that HPV vaccines cannot treat pre-existing HPV infections nor cervical cancer itself. For these reasons, the public health benefits of recent HPV vaccinations will only be evident in several decades [5]. In addition, vaccination alone is unlikely to lead to cervical cancer eradication. Therefore, vaccination should be complemented with screening to detect treatable pre- or early-stage cancer before it enters advanced stages [5].

Traditionally, cervical cancer screening is done by cytology, where a physician, gynaecologist, or other trained sampler (i.e. nurse or midwife) collects a sample of cervical cells (commonly called a Pap test) and evaluates it for the presence of cell abnormalities under a microscope. This method was introduced in 1941 and is credited with achieving a 70% reduction in cervical cancer rates in the USA [33]. However, processing cytological tests is highly dependent on a sufficient number of trained health providers to collect samples, and having access to sophisticated laboratory equipment and highly qualified pathologists to interpret the results. A number of quality concerns can be triggered when laboratories process either too few tests annually to maintain their skills or overload technicians with too many tests, both risking diagnostic errors [20]. Moreover, interpreting the test is time consuming and inherently subjective, with limited reproducibility and sensitivity to detect pre-cancer [34]. Some women might also require a second consultation if test results are atypical, undefined, to conduct further testing or begin treatment.

In places unable to support high-quality cytology, visual inspection of the cervix with acetic acid, by a trained health provider, is a low-cost and simple alternative often recommended [5]. Indeed, alternative approaches to cervical screening in resource-constrained settings have been adopted including screening women once in a lifetime using visual inspection with acetic acid (VIA) or HPV testing, which has been found to reduce lifetime risk of cancer by approximately 30% and cost less than US$500 per year of life saved [35]. Visual inspection-based screening looks for a colour change of the cervix when acetic acid is applied. While the sensitivity of VIA can be improved, the low specificity also leads to many false positives and over treatment [36]. Therefore, ensuring access to quality services remains a major barrier to scaling-up universal screening programmes based on cytology or visual inspection in many LMICs.

Now, new technologies to detect HPV DNA offer a number of advantages over cytology, which can reduce disparate access to cervical cancer screening. An HPV test uses a sample of cells from a woman’s vagina/cervix to detect the presence of high-risk subtypes of HPV DNA that increase her risk of cervical cancer. It is equally effective and a more sensitive strategy than cytological evaluation alone [37, 38]. The WHO’s 2013 Guidelines for screening and treatment of cervical cancer endorse HPV tests if the programme has sufficient resources, promoting cytology only if it meets quality indicators [39]. The 2015 European Guidelines for Quality Assurance in Cervical Cancer Screening recommend the implementation of HPV tests as a primary screening strategy [37]. Although most HPV DNA tests still require laboratory infrastructure, some new devices make it possible to bring the lab to the patient in a single, handheld test (discussed further below) [40].

### Modern screening methods to overcome disparities

NCSPs offering screening services and evidence-based technologies aligned with human rights, technological advances and modern clinical practice, and fit with the needs of both the general population and hard-to-reach populations of women, have the highest potential to achieve more [18]. Having multiple screening methods and follow-up strategies within a NCSP to increase coverage and continuity of care, respectively, is therefore an alternative that more programmes should consider. Cytology is still the most common national cervical cancer screening method, despite the difficulty implementing them in low-resource settings (i.e. without trained gynaecologists and pathologists, and laboratory facilities) [41]. In this regard, HPV DNA testing offers several advantages over cytology for expanding screening with limited resources to hard-to-reach populations.

First, HPV DNA testing allows women to take self-samples (i.e. to collect cells from herself using a vaginal swab), which is not compatible with cytology. Self-sampling is associated with higher screening coverage, particularly among vulnerable populations facing linguistic, cultural, geographic, or economic barriers [42–44]. It is also highly accepted by women because it is more discrete and less invasive than physician-obtained samples [45]. Furthermore, self-sampling “has the potential to further empower women to collect their own samples in privacy giving them control over how and when they participate in screening” [46]. HPV test results obtained in self-sampled material are highly concordant with those obtained by physicians [47–51]. Self-sampling is a strategy to increase screening participation, particularly among hard-to-reach women, because it can be safely and effectively done with support from community health workers (instead of physicians or gynaecologists) or by women alone [48, 52].

Second, screening by self-sampling in populations of hard-to-reach women can be further enhanced if used together with a user-friendly HPV rapid testing device. One of the remaining limitations of HPV testing is its reliance on laboratory infrastructure to process the results. However, HPV rapid testing devices can alleviate this constraint by bringing a portable molecular DNA test to women. Some of the present authors are part of the international research consortium, ELEVATE, which recently launched a five-year project financed by the European Commission’s Horizon 2020 programme to develop a new test and screening approach for cervical cancer in hard-to-reach women. The test will combine self-sampling with a new low-cost, portable measurement device that will be validated in screening trials in Belgium, Brazil, Ecuador, and Portugal. The new ELEVATE HPV test will yield easy-to-understand results in low-resource settings lacking specialist health personnel or electricity in remote locations. Point-of-care results mean that women can be screened and receive their results in the same visit, resulting in increased continuity of care and efficient follow-up processes. How such a device can be an added value to under-screened women is highly context dependent (i.e. home-based self-sampling, community mobilisation for testing, testing followed by clinician counselling, or in other combinations) [53]. Therefore, the ELEVATE project will also execute pilot studies in hard-to-reach populations to determine the feasibility, user and health provider acceptability, costs, logistics, and population compliance of self-sampling and the rapid HPV testing device [54, 55].

The budget impact of the HPV test has been a barrier to its widespread introduction in some LMICs. Nevertheless, NCSPs should consider a self-sampling and HPV testing method to reach under-screened women, remembering that governments may need to devote “greater resources to [these] traditionally neglected groups” to eliminate systemic discrimination and ensure their right to SRH (4, para 31). The cost-effectiveness of HPV testing methods has been shown in various contexts, while maintaining or even improving effectiveness compared to traditional cytology programmes [56]. Studies on primary HPV testing, including self-sampling methods, followed by triage for HPV-positive cases are also shown to be cost-efficient and effective in Brazil [57]. In Canada, for example, self-testing in rural populations, when combined with community engagement and education, is effective at increasing coverage in underserved populations and is a cost effective alternative [52].

## Conclusion

The crux of cervical cancer control rests in prevention through vaccination and early detection through screening. Until now, many NCSPs have been unresponsive to important social inequalities that marginalise some groups of women, hampering universal access to screening services. Under-screened women have a higher burden of cervical cancer and worse survival rates than regularly-screened women. Yet, there is good reason for Dr. Tedros to call cervical cancer “a NCD we can overcome” [2]. Governments have human rights obligations to refocus screening policies and programmes on women who are disproportionately affected by discrimination that impairs their full enjoyment of the right to SRH (4, paras 30–31). To reach underserved women, NCSPs also rely on and can contribute to strengthening the six building blocks of health systems: (1) innovative approaches to health services, such as screening through mobile health units and community health workers; (2) health information systems to manage immunisation and screening records, and community outreach in a language and manner that is *acceptable* to the target population; (3) an adequate number of trained healthcare providers to immunise, screen/sample, and interpret test results; (4) regular supply of self- sampling and point-of-care HPV testing devices that are *acceptable* to marginalised women and health providers, and of HPV vaccines (from a temperature-assured supply chain) that are *available* at an *affordable* price; (5) adequate financing for the foregoing measures; and (6) leadership and good governance to implement these measures through out the health system with particular attention for hard-to-reach populations [58]. NCSPs should tailor screening methods to under-screened women, bearing in mind that eliminating systemic discrimination may require “devoting greater resources to traditionally neglected groups” (4, para 31). NCSPs that keep these human rights principles central will be able to expand screening among low-income, isolated and other marginalised populations, but also women in general, who, for a variety of reasons, do not visit healthcare providers for regular screenings. With so much political will and global momentum towards eliminating cervical cancer as part of NCD control, the time is right to invest in evidence-driven, rights-based, innovative screening practices that target underserved women globally.

## Data Availability

Not applicable.

## Abbreviations

FDA: US Food and Drug Administration
HPV: Human papillomavirus
ICESCR: International Covenant on Economic, Social, and Cultural Rights
LMICs: Low- and middle-income countries
NCD: Non-communicable diseases
NCSP: national cervical cancer screening programmes
SRH: Sexual and reproductive health
UN CESCR: United National Committee on Economic, Social, and Cultural Rights
WHO: World Health Organization
